# Similarities between plant traits based on their connection to underlying gene functions

**DOI:** 10.1371/journal.pone.0182097

**Published:** 2017-08-10

**Authors:** Jan-Peter Nap, Gabino F. Sanchez-Perez, Aalt D. J. van Dijk

**Affiliations:** 1 Applied Bioinformatics, Wageningen University & Research, Droevendaalsesteeg 1, PB Wageningen, The Netherlands; 2 Laboratory of Bioinformatics, Wageningen University & Research, Droevendaalsesteeg 1, PB Wageningen, The Netherlands; 3 Biometris, Wageningen University & Research, Droevendaalsesteeg 1, PB Wageningen, The Netherlands; University of Guelph, CANADA

## Abstract

Understanding of phenotypes and their genetic basis is a major focus in current plant biology. Large amounts of phenotype data are being generated, both for macroscopic phenotypes such as size or yield, and for molecular phenotypes such as expression levels and metabolite levels. More insight in the underlying genetic and molecular mechanisms that influence phenotypes will enable a better understanding of how various phenotypes are related to each other. This will be a major step forward in understanding plant biology, with immediate value for plant breeding and academic plant research. Currently the genetic basis of most phenotypes remains however to be discovered, and the relatedness of different traits is unclear. We here present a novel approach to connect phenotypes to underlying biological processes and molecular functions. These connections define similarities between different types of phenotypes. The approach starts by using Quantitative Trait Locus (QTL) data, which are abundantly available for many phenotypes of interest. Overrepresentation analysis of gene functions based on Gene Ontology term enrichment across multiple QTL regions for a given phenotype, be it macroscopic or molecular, results in a small set of biological processes and molecular functions for each phenotype. Subsequently, similarity between different phenotypes can be defined in terms of these gene functions. Using publicly available rice data as example, a close relationship with defined molecular phenotypes is demonstrated for many macroscopic phenotypes. This includes for example a link between ‘leaf senescence’ and ‘aspartic acid’, as well as between ‘days to maturity’ and ‘choline’. Relationships between macroscopic and molecular phenotypes may result in more efficient marker-assisted breeding and are likely to direct future research aimed at a better understanding of plant phenotypes.

## Introduction

A major issue in current biological research is to translate differences in phenotype to variation in genotype. Detailed knowledge of phenotypes and their underlying genetics is necessary, for example for plant breeding to meet its future challenges with respect to yield, quality and production in suboptimal environmental conditions [1]. High-throughput ‘omics’ approaches generate large amounts of molecular phenotypes (transcriptome, proteome, metabolome) and new developments in high-throughput phenotyping [2, 3] are now generating increasingly large datasets of macroscopic phenotypes. Both molecular and macroscopic phenotypes are combined with genetic data using the approaches of quantitative genetics [4], resulting in a variety of quantitative trait loci (QTLs) [5–9]: eQTLs (gene expression data), mQTLs (metabolite data), and phQTLs (macroscopic data). The respective QTL types describe traits (here defined as variation in phenotype) at different levels of biological integration and complexity. A molecular phenotype can be considered to be an intermediate between the genotype and a macroscopic phenotype. The ability to reliably connect molecular traits to macroscopic traits of agronomic and/or academic interest, such as yield, heterosis or flowering time, is a next step towards better understanding and potential future use of such a trait. In maize, metabolic information helps predicting complex traits [10], and in Arabidopsis, yield, defined as biomass, is linked to specific metabolites and gene expression levels [11]. Related approaches have also been applied to study genetic correlations between human gene expression and traits [12]. Such results hold the promise of defining a molecular trait, or a combination of molecular traits, as a biomarker for a given macroscopic trait, equivalent to the use of bio(chemical)-markers for disease traits in human health research [13]. Therefore, a better connection between macroscopic traits and molecular traits is warranted.

We here present a next level analysis of the information contained in QTL regions aiming at establishing such connections. The gene information contained in QTL regions is converted to Gene Ontology (GO) terms for gene function, notably the molecular function and biological process sub-ontologies [14]. Molecular function (MF) defines the biochemical activity of a gene product, without specifying where or when the activity actually occurs. An example of an MF term is ‘adenylate cyclase’. Biological process (BP) refers to a biological concept to which a gene product contributes, usually via one or more ordered assemblies of molecular functions. An example of a BP term is ‘floral organ development’. We previously demonstrated how macroscopic traits can be linked to underlying BP ontology terms with overrepresentation analysis using QTL data [15]. The approach combined information from multiple QTL regions for the same trait and resulted in a statistically significant set of connections between traits and gene functions. This enabled prediction of QTL candidate genes which were validated by comparison with known causal genes underlying QTLs [15].

Here, we investigate and show how to compare and integrate macroscopic and molecular traits. To do so, we make use of semantic similarity [16], which is a powerful method to quantify the similarity between different sets of gene functions. The semantic similarity of lists of gene function (GO) terms that are enriched across multiple QTL regions for each trait, indicates similarity between different traits. This way, connections between traits are established. Some connections confirm or validate existing biological knowledge, whereas other connections reveal novel relationships between different (types of) traits. Such novel relationships will guide future research into understanding and exploiting macroscopic traits. Moreover, they are likely to help defining molecular traits as future proxies or markers for macroscopic traits of interest in either plant research or plant breeding.

## Results

### General approach

**Fig 1:**
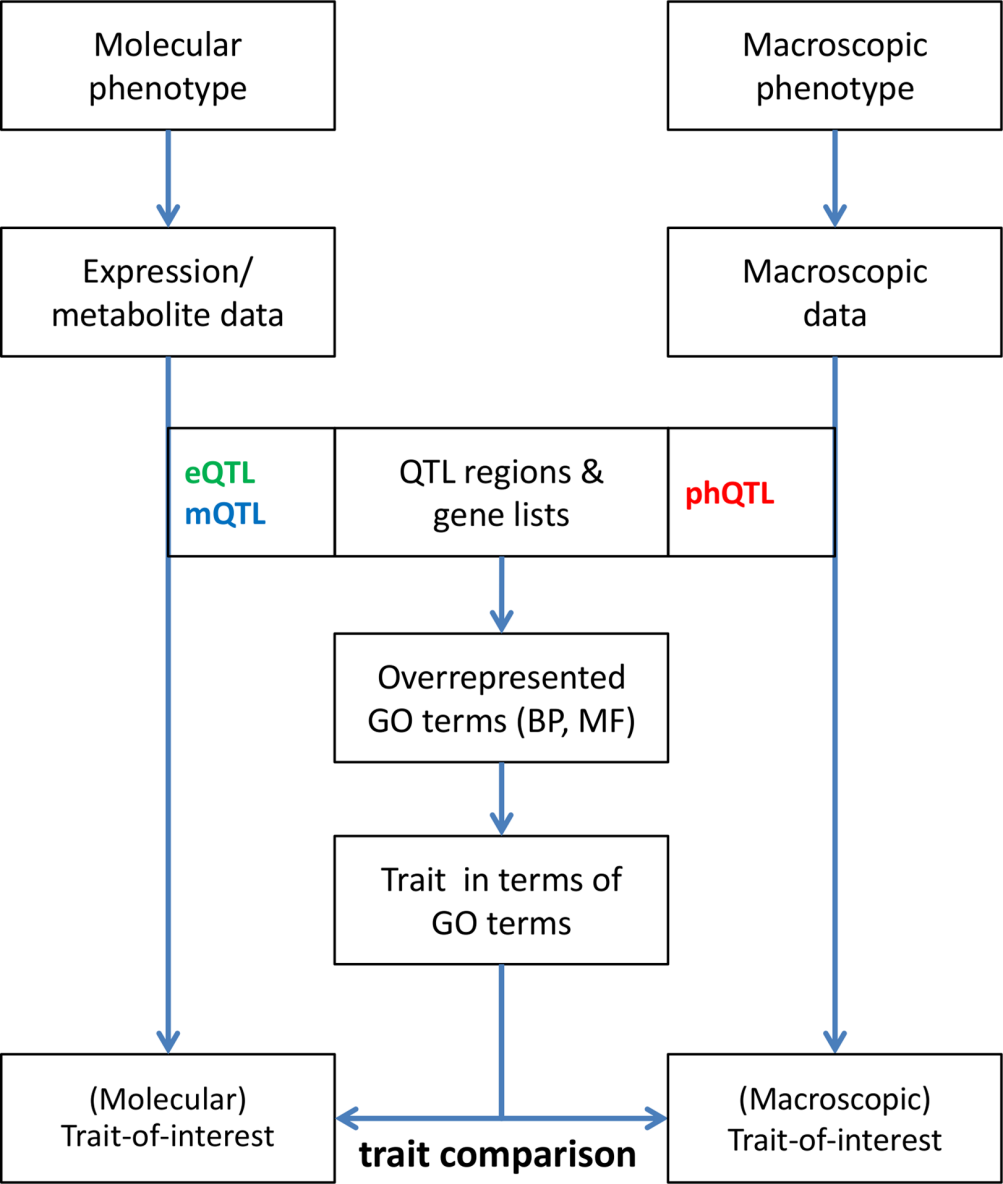
Flowchart of comparative trait analysis. Any trait of interest that is associated with multiple QTL regions, be it molecular or macroscopic in nature, is converted to a list of (overrepresented) GO terms for both biological processes (BP) and molecular functions (MF). The gene lists in the respective QTL regions are used for the prediction of GO terms and subsequent overrepresentation analysis relative to the occurrence of GO terms in the whole genome. The resulting lists of overrepresented GO terms enable comparing different traits for (dis)similarity. The formal specification of traits in GO terms allows comparison of any trait to any other trait. The figure emphasizes the comparison of molecular with macroscopic traits.

The analysis requires QTL data for different types of traits (Fig 1). QTLs associate each trait to one or more genomic regions of the crop of interest. We here focus on traits with more than one QTL region. Given multiple QTL regions for a given trait, the assumption of our approach is that there might be functional similarity between underlying causal genes for that trait in its different QTL regions. In other words, related gene functions are assumed to play a role in multiple different QTL regions for any given trait. To analyze this, the genes present in the set of QTL regions for a trait are extracted from the available structural annotation of the genome. For all these genes, the associated biological process (BP) and molecular function (MF) terms are predicted (see Methods). Overrepresentation analysis of these gene function terms characterizes the trait and establishes a connection between gene function terms and traits. Importantly, this approach combines information from multiple QTL regions for a given trait. Connections between BP terms and macroscopic traits were previously successfully used as a step to prioritize candidate genes in QTL regions [6]. Here, we establish connections between traits and gene function terms (either BP or MF). These connections allow to compare traits on the basis of the semantic similarity of their associated gene function terms.

### Connecting different traits with GO terms using public rice data

To demonstrate the novelty and added value of the approach, we used public domain data for rice (*Oryza sativa)* for macroscopic phenotype (phQTL) [17, 18], metabolite (mQTL) [19] and expression (eQTL) [5] traits. For inclusion in the analysis, it is required that a trait is associated with at least two (phQTL, mQTL) or three (eQTL) QTL regions. The latter requirement reduced the number of traits in the eQTL dataset from 2,113 to a better manageable 418. The phQTL data set contains 148 traits and the mQTL dataset 623. For all traits, the genes in the associated QTL regions were extracted from the rice genome. Various descriptive statistics of the three datasets are summarized in Table 1. The average number of QTL regions per trait, given the threshold used, ranges from 3 to 11. The average number of genes per QTL region ranges from 140 to 191, and the average number of genes associated with each trait ranges from 477 to 1,248.

**Table 1:** Descriptive statistics of datasets used^a^.

Number of:	Macroscopic traits	Metabolite traits	Expression traits
**- traits**	148	623	418
**- QTL regions per trait**	11 (14)	3 (1)	3 (1)
**- genes per trait**	1,248 (1,869)	570 (271)	477 (183)
**- genes per QTL region**	140 (121)	191 (99)	146 (91)
**- BP terms per trait**	1,106 (513)	958 (326)	1,079 (201)
**- BP terms per QTL region**	494 (344)	632 (242)	580 (255)
**- MF terms per trait**	259 (220)	162 (72)	172 (46)
**- MF terms per QTL region**	70 (49)	92 (35)	77 (36)

^a^Three different rice QTL datasets were used, containing macroscopic traits (phQTL), metabolic traits (mQTL) and expression traits (eQTL). For genes in the QTL regions for these traits, gene function terms were predicted, defined as biological process (BP) or molecular function (MF) terms. Values represent the total number or average (standard deviation).

For all genes, biological process (BP) terms and molecular function (MF) terms were predicted using BMRF [20–22] for BP terms and Argot2 [23] for MF terms. The average number of BP terms per trait ranges from 958 to 1,106, and that of MF terms from 162 to 259; the average number per QTL region ranges from 494 to 632 for BP and from 70 to 92 for MF (Table 1). The fact that the number of BP terms is several times larger than the number of MF terms is in line with what is observed for the whole genome: when considering all genes, the number of gene-GO associations is roughly four times higher for BP than for MF (S1 Table).

As previously demonstrated for BP terms in the context of QTL candidate gene prioritization for macroscopic traits, overrepresentation analysis of gene function terms allows detailed characterization of traits [6]. This procedure combines information from multiple QTL regions by requiring that an overrepresented gene function occurs in the majority of these regions. For example, the trait ‘root volume’ in the macroscopic trait dataset is associated with 5 QTL regions, in which 1,084 genes are annotated. These 1,084 genes give rise to the prediction of 1,543 BP terms, of which 17 are overrepresented and pass the threshold on occurrence in at least 3 QTL regions. This establishes the link between the trait ‘root volume’ and 17 gene function terms. This way, 130 traits in the macroscopic trait (phQTL) dataset are associated with on average 16 BP terms per trait, involving 830 unique BP terms; 102 traits are associated with on average 11 MF terms per trait, involving 560 unique MF terms (Table 2). For the macroscopic traits, this results in 2,047 links between traits and BP terms, and 1,141 links between traits and MF terms. For the other two datasets, the equivalent numbers are given in Table 2. All links established between traits and BP or MF terms are given in S2 Table.

**Table 2:** Number of links between traits and gene function terms^a^.

Number of:	Macroscopic traits	Metabolite traits	Expression traits
**- traits**	148	623	418
**- links per trait with BP**	16 (14)	17 (15)	20 (17)
**- traits involved**	130	620	406
**- BPs involved**	830	1,278	1,093
**- trait-BP links**	2,047	10,760	8,242
**- links per trait with MF**	11 (14)	23 (19)	8 (5)
**- traits involved**	102	619	399
**- MFs involved**	560	1,392	208
**- trait-MF links**	1,141	14,285	3,091

^a^Traits were linked to gene function terms on the basis of overrepresentation of biological process (BP) terms or molecular function (MF) terms for genes associated with the trait using multiple QTL regions for a given trait. Values represent the total number or average (standard deviation). The trait-gene function links themselves are presented in S2 Table.

### Biological interpretation of gene functions linked to traits

**Fig 2:**
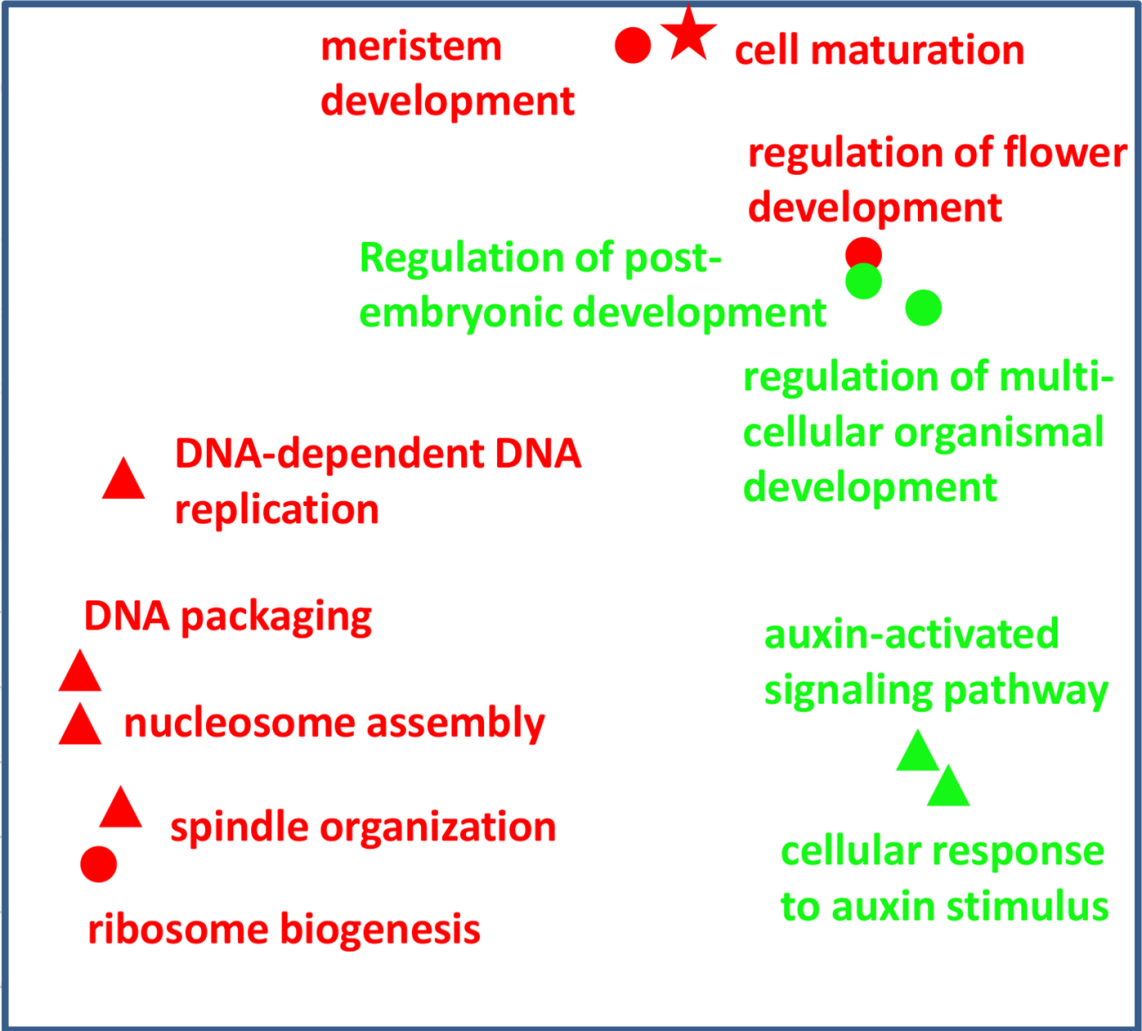
Connections with different biological process terms for three macroscopic traits (red) and two expression traits (green). Macroscopic traits included are ‘root volume’ (red triangles), ‘days to heading’ (red circles) and ‘days to maturity’ (red star). Expression traits are LOC_Os01g10504 (green circles) and LOC_Os04g30210 (green triangles). At each symbol, the biological process (BP) term connected to that trait is given. REVIGO [44] was applied to obtain a plot in which the distance between symbols indicates dissimilarity between BP terms. In this plot, the x- and y-axis do not have a direct meaning, but for each point, the x- and y-coordinates are chosen so that terms with a high semantic similarity are close to each other in the plot.

In all three datasets, many of the links between gene function terms and traits confirm and/or strengthen prior understanding of the molecular basis of traits. For three traits in the phQTL dataset, ‘days to heading’, ‘days to maturity’ and ‘root volume’, examples of links with BP terms are shown in Fig 2. In this plot, for each BP term, its position is chosen such that more similar terms are more close.

Links with the developmental trait ‘days to heading’ were presented previously [6]: the BP term ‘regulation of flower development’ is clearly part of current knowledge with respect to this trait. For the anatomical trait ‘root volume’ links with terms such as ‘DNA packaging’, ‘DNA-dependent DNA replication’ and ‘nucleosome assembly’ confirm the known connection between nuclear ploidy level and cell-size control [24]. It is also not surprising that the term ‘cell maturation’ is linked to the trait ‘days to maturity’.

Although fewer connections with MF terms are found for the traits from the phQTL dataset, also here known connections between macroscopic traits and MF terms are apparent. For example, the abiotic stress trait ‘relative shoot elongation under submergence’ is linked to two MF terms, both describing ‘DNA topoisomerase activity’. The role of topoisomerase in plant growth and development, including cell elongation, is well established [25].

In the mQTL dataset, ‘succinate, malate and citrate transport’ terms are linked to the amino acid traits ‘glutamine’ and ‘leucine’. The connection between such transporters and amino acids agrees with the knowledge that amino acid biosynthesis uses intermediates from the citric acid cycle [26]. The MF term ‘sodium:dicarboxylate symporter’ is linked to two amino acids (glutamate and leucine) and two metabolites with unknown identity. Such a symporter is known to be involved in amino acid transport [27].

The BP terms overrepresented in the eQTL regions for the expression of the gene LOC_Os01g10504 (OsMADS3) in the eQTL dataset include ‘regulation of post-embryonic development’ and ‘regulation of multicellular organismal development’ (Fig 2). OsMADS3 is a transcription factor involved in regulating processes such as flower development. For the trait LOC_Os04g30210, two of the BP terms obtained via overrepresentation analysis of its eQTL regions are related with auxin (Fig 2). The gene encodes an F-box protein, and F-box proteins are known to be involved in auxin signalling [28].

### Comparison between different traits: numbers of links with BP and MF

**Fig 3:**
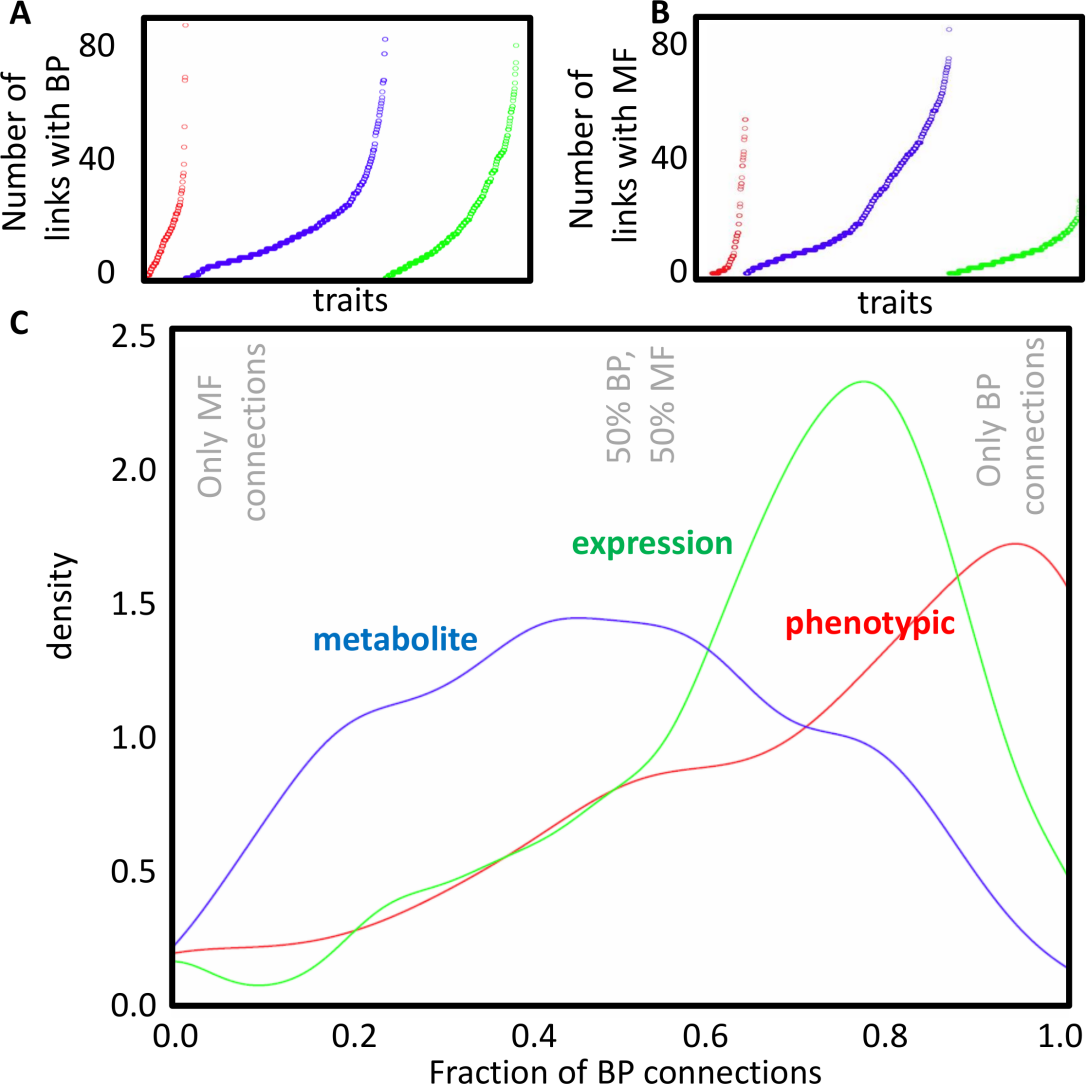
Distribution of the number of connections between traits and gene function terms. **(A-B)** The number of links with GO terms for different traits. Macroscopic traits (red), metabolite traits (blue) and expression traits (green) are placed consecutively along the horizontal axis. Within each category, traits were ordered (left to right, low to high) based on the number of connections with BP terms (A) or MF terms (B). **(C)** Distribution of the fraction of BP connections (number of BP connections/(number of BP connections + number of MF connections), for macroscopic traits (red), expression traits (green), and metabolic traits (blue). As indicated with the grey legend in the figure, a fraction of BP connections of 0 corresponds with only MF connections, and a fraction of BP connections of 1 corresponds with only BP connections.

The results presented in Table 2 and details given in S2 Table show marked differences for different types of traits. For the macroscopic traits, the average number of links obtained with BP terms is higher than the average number of links obtained with MF terms (16 for BP, 11 for MF). For expression traits, this difference is even higher (20 for BP, 8 for MF). In contrast, for metabolite traits more links with MF terms than with BP terms are obtained (23 for MF, 17 for BP). To further investigate this difference, the distribution of the number of links over the different traits was analyzed. This confirmed the difference between in particular expression traits and metabolite traits with respect to MF (Fig 3A and 3B). For further analysis, we defined the fraction of BP term links per trait (f_BP_): f_BP_ equals the number of links with BP terms obtained for a given trait, divided by the total number of links with both BP and MF terms obtained for that trait. Traits with mainly BP term links will have f_BP_ close to 1, whereas traits with mainly MF links will have values close to zero; a value of f_BP_ = 0.5 indicates that a trait has the same number of links with BP terms as with MF terms. Whereas the f_BP_ for metabolite traits is distributed over the whole range of values (Fig 3C, blue line), the traits in both the expression (green line) and macroscopic (red line) datasets tend to be more connected to BP terms: the distributions are shifted to the right in Fig 3C. A Kolmogorov-Smirnov test confirms that the three distributions are significantly different from each other.

### Comparison between different traits: semantic similarity

To assess similarity of traits, semantic similarity [16] between sets of overrepresented GO terms obtained for individual traits was calculated. This analysis was performed separately for BP and MF terms. The complete list of semantic similarities of traits based on the overrepresented GO terms they are connected with is presented in S3 Table.

A first assessment of the semantic similarities between traits focuses on cases where the semantic similarity equals one: the list of gene function terms (either BP or MF) for two different traits is identical. In total, 69 such cases were obtained (Table 3; S4 Table). For example, for each pair of five pairs of metabolite traits, the same MF terms were obtained. For three of these pairs, the identity of both metabolites in the trait is known, and in two cases these are biochemically closely related: the phospholipids LPC(1-acyl 18:1) and LPC(1-acyl 18:2), and the two flavonoids C-pentosyl-apigenin O-rutinoside and 3', 4', 5'-dihydrotricetin O-hexosyl-O-hexoside. The third pair consists of 1-O-palmitoylhexitol and pregna-5,20-dien-3-ol, which are both classified as “other” in the metabolite trait dataset. Because similar metabolites are associated with identical sets of GO terms, the approach here presented may aid in the identification of metabolites. For two of the metabolite pairs with semantic similarity equal to one, only one of the metabolites has a known identity, in both cases a terpene (Phytocassane A, and Ephemeranthoside). The GO term similarity analysis as here presented suggests that the paired metabolites with unknown identity (m0323-S and m0615-L) are terpenes as well.

**Table 3:** Semantic similarity between different types of traits^a^.

	Semantic similarity = 1^b^		Significant links^c^	
Trait type combination	BP	MF	BP	MF
**Macroscopic–Macroscopic**	1	9	79	73
**Macroscopic–Metabolic**	0	10	532	608
**Macroscopic–Expression**	0	14	368	444
**Metabolic–Metabolic**	6	5	493	488
**Metabolic–Expression**	1	6	847	892
**Expression–Expression**	3	14	312	330
**Total**	11	58	2,631	2,835

^a^For each trait, semantic similarity was calculated with other traits, using either biological process (BP) terms or molecular function (MF) terms, associated with these traits.

^b^Semantic similarity = 1 implies identical sets of gene function terms associated with both traits.

^c^For each trait, the trait with maximum semantic similarity was identified over all macroscopic, metabolic and expression traits separately. Based on randomization, the significance of the link was assessed. Values represent the total number of significant trait-trait links per category.

**Fig 4:**
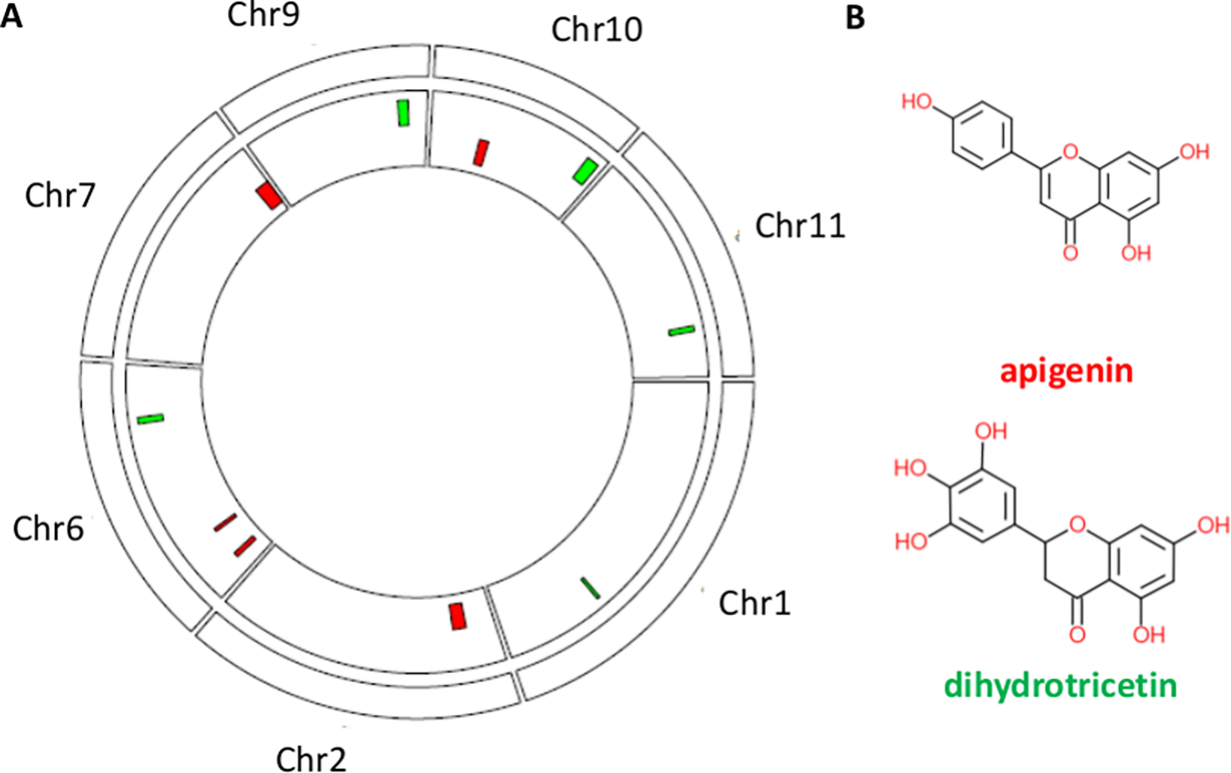
Metabolite traits with different QTL regions are associated with the same two MF terms. **(A)** QTL regions of metabolite traits C-pentosyl-apigenin O-rutinoside (red) and 3’,4’,5’-Dihydrotricetin O-hexosyl-O-hexoside (green). It is clear that the QTL regions are completely different. Both traits are however predicted to be connected to the same MFs. **(B)** Chemical structures of scaffold of the two metabolites, apigenin and dihydrotricetin. The scaffold of both compounds is very similar.

If two traits have similar QTL regions, they are more likely to be associated with the same GO terms. However, 21 of the 69 trait pairs with identical GO terms have completely different QTL regions associated with the two traits in a pair. An additional 20 cases have only one QTL region which overlaps between the two traits in a pair. Because many pairs of traits have no or only limited similarity between their QTL regions, analyses of semantic similarity allow to integrate and compare traits at a higher level than just the similarity of their QTL regions. This also holds true for these five metabolite pairs with semantic similarity one mentioned above: for three of the five pairs of metabolite traits, there is no overlap or only a very limited overlap between the QTL regions. One example is the above mentioned pair of flavonoids, C-pentosyl-apigenin O-rutinoside and 3', 4', 5'-dihydrotricetin O-hexosyl-O-hexoside (Fig 4). Both are associated by overrepresentation analysis with exactly the same two MFs that both describe serine type (endo)peptidase activity. Proteolysis by the ubiquitin/proteasome system is known to regulate flavonoids [29, 30]. The five QTL regions for C-pentosyl-apigenin O-rutinoside are completely different from the five QTL regions for 3', 4', 5'-Dihydrotricetin O-hexosyl-O-hexoside (Fig 4A). Nevertheless, the fact that they are associated to exactly the same MFs indicates their relatedness, which as indicated above is in line with the chemical similarity between these compounds.

The significance of the similarities obtained between traits was analyzed with a permutation test. As mentioned above, 69 pairs of traits were obtained with a similarity of 1; according to the permutation analysis this was significant with p = 0.002. Similarly, the number of trait pairs with a semantic similarity of at least 0.95 or 0.9 was also found to be significant (p = 0.001 in both cases).

### Linking traits to each other: maximum semantic similarity

**Fig 5:**
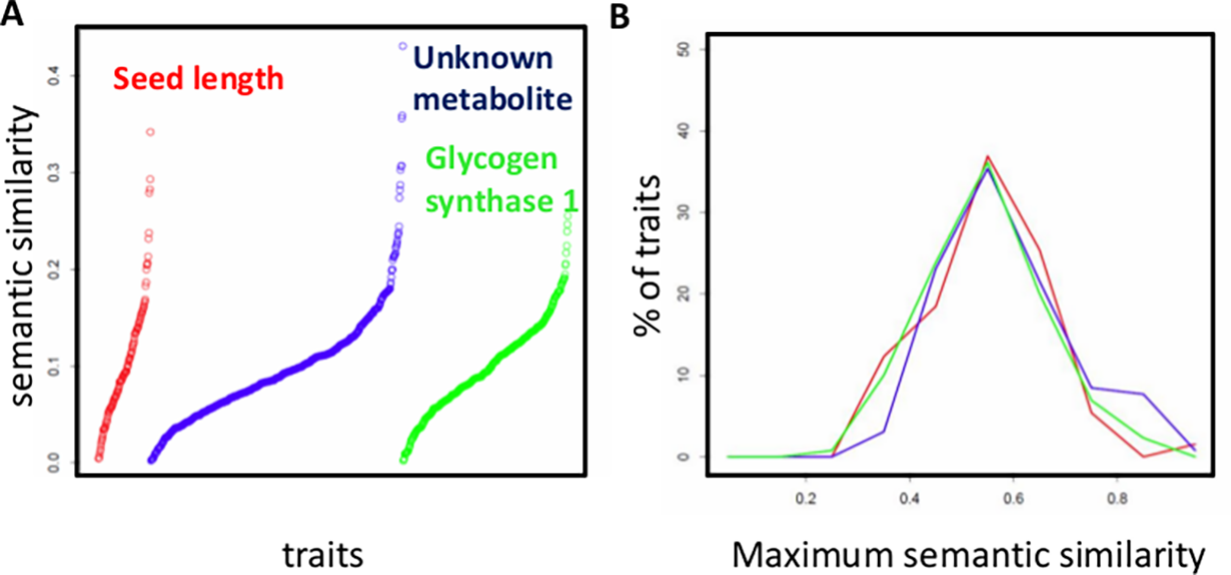
Semantic similarity allows comparisons of traits. **(A)** Semantic similarity based on BP terms for the macroscopic trait **‘**days to maturity’ with other macroscopic traits (red), metabolite traits (blue) and expression traits (green). Traits are ordered based on their semantic similarity to ‘days to maturity’ (left to right, low to high). For each category of traits, the name of the trait with maximum semantic similarity to ‘days to maturity’ is given. **(B)** Distribution of maximum semantic similarity based on BP terms for macroscopic traits with other macroscopic traits (red), metabolic traits (blue), and expression traits (green).

For any given trait, its semantic similarity with other traits displays a range of values. The semantic similarity values based on overrepresented BP terms for the trait ‘days to maturity’ relative to all macroscopic traits, metabolite traits and expression traits are plotted in Fig 5A. It shows that most traits have a relatively low semantic similarity to ‘days to maturity’. Ordering traits based on semantic similarity allows identification of the trait with the maximum semantic similarity to ‘days to maturity’. In this case the macroscopic trait with maximum semantic similarity (of 0.34) is ‘seed length’, the metabolic trait with maximum semantic similarity (of 0.43) is a metabolite of unknown identity and the expression trait with maximum semantic similarity (of 0.26) is LOC_Os02g56320.

This way, for each trait, the maximum semantic similarity with all other traits was obtainedseparately for macroscopic, metabolic and expression traits. The distribution of the maximum semantic similarity between each macroscopic trait and all other traits based on BP terms displays a range of values from ~ 0.25–1.0. This indicates that some macroscopic traits have a high semantic similarity with another macroscopic, metabolite or expression trait, whereas for other macroscopic traits, no such high semantic similarity with any macroscopic, metabolic or expression trait is observed (Fig 5B). Similar analyses based on MF and for other trait-trait combinations are presented in S1 Fig.

To analyze the significance of the maximum semantic similarity of a trait with another trait, randomized trait-GO connections were used as input to generate expected values of maximum semantic similarity between traits. All trait-trait links were filtered based on significance using a stringent multiple testing correction (see Methods for details). For example, for the three links with days-to-maturity based on BP terms mentioned above, only the link with the metabolic trait was significant. The total number of significant links between traits is given in Table 3. All links between traits based on maximum semantic similarity both for BP and MF terms are detailed in S5 Table. A Cytoscape [31] network visualization of the links between traits is provided in S1 Dataset.

### Clustering traits based on semantic similarity

**Fig 6:**
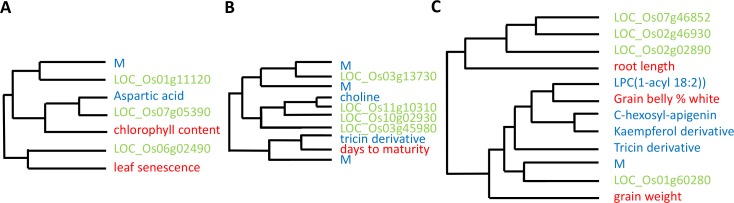
Parts of dendrograms based on semantic similarity between different traits. All dendrograms show semantic similarity relationships for different macroscopic traits (red), metabolite traits (blue) and expression traits (green). Unidentified metabolites are indicated by ‘M’. **(A)** Semantic similarity relationships for the traits ‘leaf senescence’ and ‘chlorophyll content’, based on associations with BP terms. **(B)** Semantic similarity relationships for the trait ‘days to maturity’, based on associations with MF terms. **(C)** Semantic similarity relationships for the trait ‘root length’, based on associations with BP terms.

The analysis described above was based on maximum semantic similarity to obtain pairwise links between traits (S5 Table, S1 Dataset). A more detailed view of trait-trait similarity is obtained by clustering traits based on their semantic similarity values. This allows visualizing and analyzing the similarity of several traits with respect to each other simultaneously. The resulting dendrograms contain clusters of traits at different levels of granularity (small or large clusters). Dendrograms are available in S2 Dataset, and can be visualized via http://itol.embl.de/tree/7716210613417711454683547 (BP terms) and http://itol.embl.de/tree/7716210613125231454685700 (MF terms). Three specific examples of small clusters (Fig 6) are here discussed in more detail.

(i) The macroscopic traits ‘leaf senescence’ and ‘chlorophyll content’ cluster with the metabolite trait ‘aspartic acid’ and three expression traits on the basis of semantic similarity in their BP terms (Fig 6A). For some of the relationships in this cluster, evidence exists. Both chlorophyll degradation and aspartic proteases play a role in leaf senescence [32]. The gene LOC_Os06g02490, is an acyl CoA binding protein, also a type of protein known to function in leaf senescence [33]. Other relationships indicate connections between traits that are less obvious and may point to hitherto unknown involvement of genes in these macroscopic traits. The gene LOC_Os07g05390 is a putative myosin heavy chain protein, and LOC_Os01g11120 is a putative RNA-binding protein. The semantic similarity relationships here identified suggest that these genes are involved in leaf senescence.

(ii) The macroscopic trait ‘days to maturity’ clusters with the two metabolite traits ‘tricin derivative’ and ‘choline’ (Fig 6B) on the basis of semantic similarity in their MF terms. These three traits share the MF term ‘peroxidase activity’ according to the overrepresentation analysis (S2 Table). Peroxidases are known to be involved in ‘days to maturity’ [34, 35] and the overrepresentation analysis suggests that they may be relevant for these two metabolites as well. We are not aware of any hint to a relationship between the metabolites ‘tricin derivative’ and ‘choline’ and the macroscopic trait ‘days to maturity’ in the scientific literature. Such novel relationships warrant future investigations and may shed new light on the mechanisms underlying ‘days to maturity’.

(iii) The macroscopic trait ‘root length’ clusters with the metabolite trait kaempferol and four other metabolite traits (Fig 6C) on the basis of semantic similarity in their BP terms. Kaempferol is known to influence auxin transport in roots [36] which could explain the connection between this metabolite and root length. For the other metabolites, involvement in root length is a novel prediction. Other traits clustering with these metabolites are ‘grain belly percentage white’ and ‘grain weight’ as well as the expression of four genes (Fig 6C).

These examples demonstrate that in addition to similarity relationships that confirm and/or validate biological knowledge, other similarity relationships are new or unexpected and may point to potentially new biological insights. Such new relationships illustrate how analyses on the basis of semantic similarity can provide potential molecular biomarkers for macroscopic traits and are likely to improve the understanding of the biological mechanism of the traits.

## Discussion

The basis assumption underlying the overrepresentation approach is that in several of multiple QTL regions for a given trait, a causal gene is present, and that several causal genes have related or similar gene functions. We have demonstrated that macroscopic, metabolite and expression traits with multiple QTL regions in the crop rice can all be analyzed for enrichment of GO terms and all result in lists of significantly overrepresented GO terms.

GO terms are an abstract representation of gene function, either in terms of a biological process (BP) or a molecular function (MF). The different trait types differ considerably in the number of overrepresented terms they link to. The number of links between metabolite traits and MF terms was larger than with BP terms, whereas the macroscopic traits linked more with BP terms. This difference may indicate that variation in macroscopic traits is predominantly influenced by biological processes (i.e. biological concepts to which various molecular functions contribute), whereas variation in metabolite levels is largely influenced by well-defined biochemical (molecular) functions such as enzymatic reactions or transport. Such activities on, or resulting in, a given metabolite, may have a direct impact on metabolite level. Expression traits are more similar to macroscopic traits: more links were obtained with BP terms. This may seem a bit surprising at first, as expression traits are also molecular traits. However, it indicates that expression traits are much more complex than the term ‘molecular trait’ may suggests. Many more processes and factors establish the variation in expression than well-defined molecular functions can describe.

The GO term overrepresentation analysis combines information from multiple QTL regions for a given trait to obtain links between trait and gene function terms. Traits with only one QTL region are not included in the analysis. It would however be straightforward to improve on the analysis by incorporating additional QTL data. With data from additional populations and/or different species, more traits can be included, and more accurate GO term lists will be obtained to connect and compare different traits to generate leads for future use or research.

Overrepresented GO terms for the various traits create a common ground for comparison of heterogeneous trait types. For example, a link is predicted between the metabolite ‘choline’ and ‘days to maturity’. Such a relationship may result in a biomarker at the metabolite or gene expression level. To assess the predictive value of this potential biomarker, one could e.g. measure choline metabolite levels during growth and establish whether these are correlated to the trait of ‘days to maturity’. When validated, choline may be easier and cheaper to measure than ‘days to maturity’. The determination of a macroscopic trait is generally most time- and resources consuming. Therefore translation of such a trait into an easier-to-determine molecular trait is worthwhile even without full understanding of the underlying biology. This set-up is similar to the application of molecular biomarkers for disease traits in human health [13]. In plants, the added value of the use of molecular traits as marker for macroscopic traits is being explored [10, 37, 38]. An important advantage of such new molecular markers arises if they are measured earlier than the macroscopic phenotype. This would speed up the process of selection and breeding.

The metabolite QTLs were measured in flag leafs and in seeds. Any prediction about metabolite traits is therefore restricted to their role in these tissues. More generally, the experimental conditions that were applied during the experiments to obtain the QTL regions could influence our results. Given the lack of standardized descriptions of experimental conditions [3], it is currently not possible to analyze the influence of experimental conditions in more detail. Notably the macroscopic phenotypic QTL regions are derived from a variety of sources that lack standardized metadata.

Semantic similarity based on overrepresented GO terms as here presented provides a new way of obtaining connections between different types of traits that may define novel markers for traits. The very many trait-trait relationships that do not yet translate easily in current biological models or knowledge could help direct or focus future academic or applied research. Similarity with other traits may generate hypotheses about their identity that can be experimentally investigated. For example, given that only 272 out of the 623 traits in the mQTL dataset have a known identity (characterized metabolites), the possibility to characterize unknown metabolites in terms of their similarity to known metabolites, is one of the attractive applications of the GO-term based method of linking traits here presented.

This approach is complementary to the approach of finding direct correlation between macroscopic traits measurements and metabolite levels. Such approaches have been demonstrated for various plant systems [39, 40]. Direct correlation can only be demonstrated when macroscopic traits and metabolite traits are measured in the same plant population. The large set of macroscopic trait QTL data here used comes from various populations, impeding the use of correlation to generate trait similarity networks. Moreover, the use of semantic similarity focuses on gene function terms that define similarity between traits. For example, the semantic similarity between ‘days to maturity’ and ‘choline’ was among others based on the mutual term ‘peroxidase activity’. Such information cannot be obtained with only the correlation between traits.

Overlap between QTL regions associated with gene expression and QTL regions associated with trait values has been analyzed previously as an alternative approach to assess similarity between traits [7, 41]. The focus on gene function terms allows the analysis of similarities between traits which are linked to different QTL regions. The large set of macroscopic trait data used as input originates from a set of populations. Given differences between such populations, direct overlap between QTL regions is not necessarily expected for similar traits. Even for traits with a semantic similarity of one (i.e. traits connected to the same set of gene function terms) the vast majority does not have any, or just one, overlapping QTL region.

Semantic similarity of traits does not necessarily imply causation between the molecular and macroscopic traits involved, nor does it give information about the direction of possible causality. The direction of causality and whether there is a direct or an indirect influence of one trait on another are all issues for further study. For better understanding of the underlying biology, the direction of causality is relevant. Experiments are conceivable in which metabolite levels are altered to study the effect on the macroscopic trait, or conversely, in which the macroscopic trait is perturbed for example via mutation of a relevant gene, and the effect on metabolite levels is assessed. No such detailed biological insight is necessary for the use of molecular traits as easy biomarker for macroscopic traits.

## Conclusions

By connecting gene functions to different types of traits, different biological levels can be integrated and compared on a level that was not yet possible. This approach helps to disentangle the underlying mechanisms of traits and compare different traits with each other. It will help to direct future research aimed at better understanding and use of plant phenotypes in the context of plant genotypes, and ultimately enable more efficient marker-assisted breeding programs in next-generation ‘breeding by design’ [42, 43].

## Material and methods

### QTL datasets, retrieval of QTL regions and genes

The phQTL dataset contains QTL regions for macroscopic phenotypes from various studies assembled by Gramene [17, 18]. The mQTL dataset contains QTL regions for metabolite concentrations in flag leaf at the heading date and seeds 72h after germination, from offspring of a cross between two elite indica varieties of rice, ZS97 and MH63 [19]. The eQTL dataset presents QTL regions for gene expression from leaf material from offspring from a cross between the same two indica varieties [5].

QTL intervals reported as significant in the respective publications [5, 17, 19] were used as starting point. Genes in these QTL intervals were obtained from the rice 2009-01-MSU genome build downloaded from Gramene [18]. To prevent too large regions to be used, a cutoff on the maximum number of genes for a QTL interval was set to 450 genes as previously discussed [15]. As second threshold it was required that a trait connects to at least two (phQTL, mQTL), or three, QTL regions (eQTL).

### Analysis of overrepresentation of GO terms

To predict the biological process (BP) terms for each gene, BMRF was applied as described [20–22]. Predictions of molecular function (MF) terms were obtained with argot2 [23], using default settings. For the sets of genes contained in QTL regions associated with a particular trait, the occurrence of BP or MF terms was compared with the overall occurrence of these terms in the genome. Statistical significance was assessed with Fisher exact tests and Benjamini-Hochberg multiple testing correction (FDR = 0.1) using the method presented previously [15]. To prevent the use of statistically overrepresented terms present only in a small number of QTL regions, the minimum fraction of QTL regions in which the BP or MF term should at least occur was set to 0.5. To prevent the use of terms which are overrepresented in the QTL regions for a trait but which are very general (high-level) only BP or MF terms were included which were not annotated for more than 1% of all the genes in the genome. These threshold values were optimized previously [15]. To visualize sets of GO terms, REVIGO [44] was applied.

### Semantic similarity of traits based on GO terms

Semantic similarity between traits was calculated trait-by-trait with the Gossto tool [16]. With the Integrated Similarity Measure (ISM) approach applied to the Lin semantic similarity measure [45], similarity values were obtained between pairs of GO terms. These GO-term-wise similarities were subsequently converted into trait-wise similarities in the following way. When comparing trait X with trait Y, each GO term associated with X was compared with all GO terms associated with Y, and the best match (highest similarity) was obtained. The values of all the best matches between X and Y were subsequently averaged to obtain the ‘best match average’ [46]. Significance of the similarity values was assessed using a permutation approach. All trait-GO connections were shuffled by randomly assigning GO terms to traits (keeping the same total number of traits, the same total number of GO terms, and the same number of GO connections per trait). This was repeated 1,000 times, and for each of these randomized sets of trait-GO connections, the trait-trait similarity values were calculated as above. Significance was calculated by assessing how often the experimental similarity value was higher than the values obtained in the randomization, followed by Benjamini-Hochberg multiple testing correction (FDR = 0.05).

To cluster traits based on semantic similarity values, similarity values were converted to distances by applying a linear transformation: distance = sim_max_-sim where sim is the semantic similarity value and sim_max_ the maximum observed value over all pairs of traits. Clustering based on distance between traits was performed using the R-function hclust using complete linkage [47]. Kernel density estimation was performed using the R-function density [47]. Trait networks were generated and visualized using Cytoscape [31]. Traits were connected in the network if there was a statistically significant link between them based on maximum semantic similarity. Nodes in the network were colored by trait type (expression, metabolite or macroscopic) and the network layout was set to spring embedded. Visualization of QTL regions was performed using the R-package circlize [48].

### Statistical significance of the similarities between traits

The significance of the similarities obtained between traits was analyzed with two different types of permutation tests. (i) Use of randomly chosen genome regions as input for each trait, instead of QTL regions. The number of randomized regions and their length was identical to that of the QTL regions, and 1,000 of such randomized input sets were used. The exact same procedure and settings as described above were applied to these regions to obtain trait-trait similarities: overrepresentation analysis, filtering on the minimum fraction of QTL regions in which a GO term should at least occur (0.5), filtering on the number of genes to which a GO term was annotated (not more than 1%), and semantic similarity calculation. The p-value was calculated as the number of times (out of 1,000) for which the permutated dataset resulted in at least a similar number of trait-trait pairs with a given value of semantic similarity. (ii) To analyze the significance of the trait-trait links obtained based on maximum semantic similarity of a trait with other traits, randomized trait-GO connections were used as input to generate expected values of maximum semantic similarity between traits. The p-value was calculated as the number of times (out of 1,000) for which the permutated dataset resulted in at least the same value of semantic similarity for the trait-trait connection. This was followed by Benjamini-Hochberg multiple testing correction (FDR 0.05).

## Supporting information

S1 Table Characteristics of rice gene function annotation. Rice gene function terms were predicted using BMRF for biological process (BP) terms and Argot2 for molecular function (MF) terms. The total number of annotations, the number of genes with at least one annotation, and the unique number of gene function terms involved is shown.
(DOCX)

S2 Table Links between traits and biological process (BP) and molecular function (MF) terms. The following sheets are provided:
phQTL–BP
phQTL–MF
mQTL–BP
mQTL–MF
eQTL–BP
eQTL–MF
In each sheet, links between traits and biological process (BP) terms or molecular function (MF) terms are presented. phQTL indicates macroscopic traits; mQTL indicates metabolite traits; eQTL indicates expression traits. For macroscopic traits, the corresponding Trait Ontology (TO) identifier is given. For metabolite traits, the identifier is given, and for metabolites with known identity, also the metabolite name is given. For expression traits, gene names are given.
(XLSX)

S3 Table Similarity values between all trait-trait pairs. This file contains one sheet for the semantic similarity based on BP terms, and one sheet for semantic similarity based on MF terms. Pairs of traits are ordered based on the level of semantic similarity.
(XLSX)

S4 Table Pairs of traits with semantic similarity = 1. Traits associated with exactly the same set of overrepresented BP or MF terms. Each row indicates a pair of such traits. For macroscopic traits, the Trait Ontology identifier and the trait name is given; for metabolites, the metabolite identifier is given, and for characterized metabolites, the metabolite name.
(DOCX)

S5 Table Connections between traits based on significant maximum similarity. This file contains the following sheets, presenting the most similar trait for each trait in the other set of traits:
ph–ph (BP): for each macroscopic trait, the most similar macroscopic trait, based on BP terms
ph–m (BP): for each macroscopic trait, the most similar metabolite trait, based on BP terms
ph–e (BP): for each macroscopic trait, the most similar expression trait, based on BP terms
m–ph (BP): for each metabolite trait, the most similar macroscopic trait, based on BP terms
m–m (BP): for each metabolite trait, the most similar metabolite trait, based on BP terms
m–e (BP): for each metabolite trait, the most similar expression trait, based on BP terms
e–ph (BP): for each expression trait, the most similar macroscopic trait, based on BP terms
e–m (BP): for each expression trait, the most similar metabolite trait, based on BP terms
e–e (BP): for each expression trait, the most similar expression trait, based on BP terms
ph–ph (MF): for each macroscopic trait, the most similar macroscopic trait, based on MF terms
ph–m (MF): for each macroscopic trait, the most similar metabolite trait, based on MF terms
ph–e (MF): for each macroscopic trait, the most similar expression trait, based on MF terms
m–ph (MF): for each metabolite trait, the most similar macroscopic trait, based on MF terms
m–m (MF): for each metabolite trait, the most similar metabolite trait, based on MF terms
m–e (MF): for each metabolite trait, the most similar expression trait, based on MF terms
e–ph (MF): for each expression trait, the most similar macroscopic trait, based on MF terms
e–m (MF): for each expression trait, the most similar metabolite trait, based on MF terms
e–e (MF): for each expression trait, the most similar expression trait, based on MF terms
In each of these sheets, for a given trait, the most similar trait is listed, followed by the p-value (after multiple testing correction).
(XLSX)

S1 Fig Semantic similarity between traits. Histograms of the maximum semantic similarity for each trait with any other trait of either the same type or of different type, based on either BP or MF terms. **(A)** Macroscopic traits, based on BP. **(B)** Metabolic traits, based on BP. **(C)** Expression traits, based on BP. **(D)** Macroscopic traits, based on MF. **(E)** Metabolic traits, based on MF. **(F)** Expression traits, based on MF. In all panels, red indicates maximum similarity to macroscopic traits, blue maximum similarity to metabolic traits, and green maximum similarity to expression traits.
(PNG)

S1 Dataset Networks connecting traits based on BP and on MF. In these networks, each node represents a trait, and a connection between nodes indicates a significant similarity between the two traits. The following files are provided:
NetworkBP.sif, networkMF.sif (networks based on BP and on MF, respectively, in sif format)
NodeattributesBP.out, nodeattributesMF.out (Cytoscape attributes files for nodes in the network; this describes the type of trait for each node).
These files can be used to visualize the network in Cytoscape. Two Cytoscape session files are also provided which directly provide a view of the network using a spring-embedded layout, and coloring the nodes based on the type of trait: networkBP_colorednodes.cys and networkMF_colorednodes.cys.
(ZIP)

S2 Dataset Dendrograms representing results of clustering of traits based on BP and on MF. Two files are provided: hclustBP_exported_tree.newick and hclustMF_exported_tree.newick. These can be visualized in various tree/phylogeny software tools, e.g. in figtree http://tree.bio.ed.ac.uk/software/figtree/.
A visualization of these trees is also provided via http://itol.embl.de/tree/7716210613417711454683547 (BP) and http://itol.embl.de/tree/7716210613125231454685700 (MF).
To briefly explain the use of iTOL, we will focus on reproducing Fig 6C:
Open the BP based trait clustering tree by pasting http://itol.embl.de/tree/7716210613417711454683547 in a web-browserChange the display mode from Circular to Normal (via the top right hand control)Note that trait names are now visible; green indicate expression traits, blue metabolite traits and red macroscopic traits. For macroscopic traits, hovering over the trait ontology identifier displays the trait name. Similarly, for metabolite traits with known identity, the metabolite name, the way in which the metabolite was identified, and the compound class, are indicated.Use the Tree search tool (available at the top left hand side): type 0000227, the TO identifier for root length.The result displays the part of the tree around the trait root length. Similar to Fig 6C, three gene expression traits are most closely clustered with root length. Two additional macroscopic traits (grain belly percentage white, TO 0000383 and grain weight, TO 0000589), one additional expression trait and five additional metabolite traits form a second group of closely related traits. Note that the vertical ordering of these traits can deviate from the ordering in Fig 6C; the relationships indicated by the tree are however identical.
(ZIP)
