# Supplementary material for: Similarities between plant traits based on their connection to underlying gene functions
Source: PLoS One. 2017 Aug 10;12(8):e0182097. doi: 10.1371/journal.pone.0182097 (PMC5552327; doi:10.1371/journal.pone.0182097)
Supplement: S1 Table — Rice gene function terms were predicted using BMRF for biological process (BP) terms and Argot2 for molecular function (MF) terms. The total number of annotations, the number of genes with at least one annotation, and the unique number of gene function terms involved is shown. (DOCX) [file pone.0182097.s001.docx]

**S1 Table. Characteristics of rice gene function annotation^a^**

|  | **BP** | **MF** |
| --- | --- | --- |
| **#annotations** | 898,665 | 289,105 |
| **#genes** | 38,998 | 50,931 |
| **#functions** | 1,767 | 1,880 |

^a^Rice gene function terms were predicted using BMRF for biological process (BP) terms and Argot2 for molecular function (MF) terms. The total number of annotations, the number of genes with at least one annotation, and the unique number of gene function terms involved is shown.
