# Supplementary material for: Similarities between plant traits based on their connection to underlying gene functions
Source: PLoS One. 2017 Aug 10;12(8):e0182097. doi: 10.1371/journal.pone.0182097 (PMC5552327; doi:10.1371/journal.pone.0182097)
Supplement: S4 Table — Traits associated with exactly the same set of overrepresented BP or MF terms. Each row indicates a pair of such traits. For macroscopic traits, the Trait Ontology identifier and the trait name is given; for metabolites, the metabolite identifier is given, and for characterized metabolites, the metabolite name. (DOCX) [file pone.0182097.s004.docx]

**S4 Table. Pairs of traits with semantic similarity = 1^a^**

Based on BP terms:

| m0033-S | L-Glutamic acid | m0793-S | Tricin O-rutinoside |
| --- | --- | --- | --- |
| LOC_Os03g60530 |  | LOC_Os06g39690 |  |
| m0323-S |  | m0450-S | Ephemeranthoside |
| LOC_Os06g12790 |  | m0804-L |  |
| m0273-S |  | m0943-L |  |
| TO:0000366 | reproductive growth time | TO:0000369 | vegetative growth time |
| m0631-L | LPC(1-acyl 18:2) | m0639-L | LPC(1-acyl 18:1) |
| m0160-L |  | m0963-L |  |
| LOC_Os01g74450 |  | LOC_Os03g60530 |  |
| LOC_Os01g74450 |  | LOC_Os06g39690 |  |
| m0695-L | Epicatechin O-hexoside | m0944-L |  |

Based on MF terms:

| LOC_Os01g39810 |  | LOC_Os01g40690 |  |
| --- | --- | --- | --- |
| LOC_Os02g10860 |  | LOC_Os06g39330 |  |
| LOC_Os01g12210 |  | LOC_Os06g48750 |  |
| TO:0000430 | germination speed | LOC_Os10g06720 |  |
| m0141-S |  | LOC_Os09g27970 |  |
| TO:0000502 | KClO3 resistance | LOC_Os03g16470 |  |
| LOC_Os03g45340 |  | LOC_Os04g07280 |  |
| m0212-L | Pregna-5,20-dien-3-ol | m0564-L | 1-O-Palmitoylhexitol |
| LOC_Os03g60530 |  | LOC_Os06g39690 |  |
| m0615-L |  | TO:0000586 | seminal root length |
| m0132-S | Phytocassane A | TO:0000586 | seminal root length |
| LOC_Os05g05530 |  | TO:0000547 | primary branch |
| LOC_Os02g32110 |  | TO:0000547 | primary branch |
| m0508-S | Chrysoeriol 7-O-hexoside | TO:0000547 | primary branch |
| TO:0000547 | primary branch | LOC_Os11g07450 |  |
| LOC_Os03g45340 |  | TO:0000672 | rice bran percentage |
| TO:0000672 | rice bran percentage | LOC_Os04g07280 |  |
| LOC_Os01g72860 |  | LOC_Os02g46930 |  |
| m0132-S | Phytocassane A | m0615-L |  |
| TO:0000516 | callus induction | TO:0000586 | seminal root length |
| TO:0000516 | relative root length | m0615-L |  |
| TO:0000291 | carbohydrate content | TO:0000531 | anther length |
| LOC_Os02g39700 |  | LOC_Os03g22740 |  |
| LOC_Os05g03530 |  | LOC_Os08g41850 |  |
| TO:0000366 | reproductive growth time | TO:0000369 | vegetative growth time |
| m0631-L | LPC(1-acyl 18:2) | m0639-L | LPC(1-acyl 18:1) |
| LOC_Os05g05530 |  | m0508-S | Chrysoeriol 7-O-hexoside |
| LOC_Os05g05530 |  | LOC_Os11g07450 |  |
| LOC_Os02g32110 |  | m0508-S | Chrysoeriol 7-O-hexoside |
| LOC_Os02g32110 |  | LOC_Os11g07450 |  |
| m0834-L | 3', 4', 5'-Dihydrotricetin O-hexosyl-O-hexoside | m0875-L | C-pentosyl-apigenin O-rutinoside |
| TO:0000449 | grain yield per plant | LOC_Os03g51680 |  |
| TO:0000249 | leaf senescence | TO:0000586 | seminal root length |
| TO:0000249 | leaf senescence | m0615-L |  |
| LOC_Os01g10450 |  | m0493-L | Glochidacuminoside C |
| LOC_Os01g10450 |  | LOC_Os04g52730 |  |
| m0493-L | Glochidacuminoside C | TO:0000538 | large vascular bundle number to leaf area ratio |
| LOC_Os01g10450 |  | TO:0000538 | large vascular bundle number to leaf area ratio |
| TO:0000538 | large vascular bundle number to leaf area ratio | LOC_Os04g52730 |  |
| LOC_Os01g74450 |  | LOC_Os06g39690 |  |
| m0508-S | Chrysoeriol 7-O-hexoside | LOC_Os11g07450 |  |
| m0323-S |  | m0450-S | Ephemeranthoside |
| TO:0000576 | culm length | TO:0000586 | seminal root length |
| TO:0000576 | culm length | m0615-L |  |
| TO:0000344 | days to flower | LOC_Os07g04910 |  |
| TO:0000224 | iron sensitivity | LOC_Os07g04910 |  |
| TO:0000224 | iron sensitivity | TO:0000344 | days to flower |
| LOC_Os05g03530 |  | TO:0000227 | root length |
| TO:0000227 | root length | LOC_Os08g41850 |  |
| m0493-L | Glochidacuminoside C | LOC_Os04g52730 |  |
| TO:0000516 | relative root length | m0132-S | Phytocassane A |
| TO:0000516 | relative root length | TO:0000576 | culm length |
| LOC_Os02g32110 |  | LOC_Os05g05530 |  |
| TO:0000249 | leaf senescence | m0132-S | Phytocassane A |
| TO:0000249 | leaf senescence | TO:0000516 | relative root length |
| TO:0000249 | leaf senescence | TO:0000576 | culm length |
| LOC_Os01g74450 |  | LOC_Os03g60530 |  |
| TO:0000576 | culm length | m0132-S | Phytocassane A |

^a^Traits associated with exactly the same set of overrepresented BP or MF terms. Each row indicates a pair of such traits. For macroscopic traits, the Trait Ontology identifier and the trait name is given; for metabolites, the metabolite identifier is given, and for characterized metabolites, the metabolite name.
