# Supplementary figures and images for: Similarities between plant traits based on their connection to underlying gene functions
Source: PLoS One. 2017 Aug 10;12(8):e0182097. doi: 10.1371/journal.pone.0182097 (PMC5552327; doi:10.1371/journal.pone.0182097)

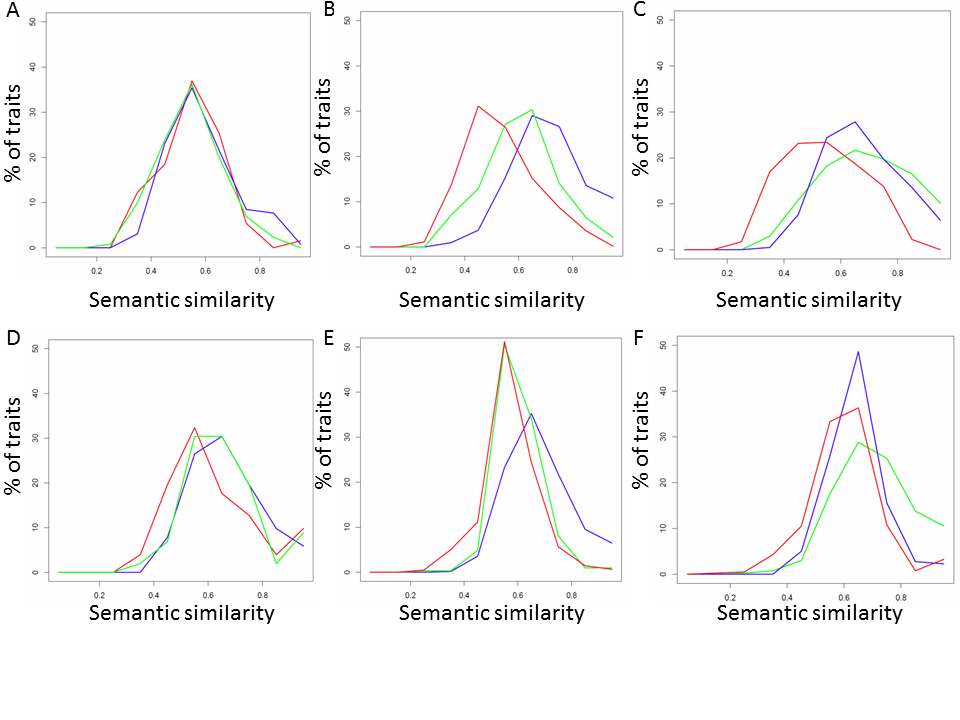

Supplement: S1 Fig — Histograms of the maximum semantic similarity for each trait with any other trait of either the same type or of different type, based on either BP or MF terms. (A) Macroscopic traits, based on BP. (B) Metabolic traits, based on BP. (C) Expression traits, based on BP. (D) Macroscopic traits, based on MF. (E) Metabolic traits, based on MF. (F) Expression traits, based on MF. In all panels, red indicates maximum similarity to macroscopic traits, blue maximum similarity to metabolic traits, and green maximum similarity to expression traits. (PNG) [file pone.0182097.s006.png]
